# Macrophage colony-stimulating factor and its role in the tumor microenvironment: novel therapeutic avenues and mechanistic insights

**DOI:** 10.3389/fonc.2024.1358750

**Published:** 2024-04-04

**Authors:** Li Yi, Yihan Gai, Zhuo Chen, Kecan Tian, Pengfei Liu, Hongrui Liang, Xinyu Xu, Qiuyi Peng, Xiaoqing Luo

**Affiliations:** ^1^ Medical Technology College of Qiqihar Medical College, Qiqihar, Heilongjiang, China; ^2^ School of Stomatology, Qiqihar Medical College, Qiqihar, Heilongjiang, China; ^3^ School of Basic Medical Sciences, Qiqihar Medical College, Qiqihar, Heilongjiang, China

**Keywords:** macrophage colony-stimulating factor, tumor progression, CSF-1R, immune modulation, dendritic cells, cancer therapy

## Abstract

The tumor microenvironment is a complex ecosystem where various cellular and molecular interactions shape the course of cancer progression. Macrophage colony-stimulating factor (M-CSF) plays a pivotal role in this context. This study delves into the biological properties and functions of M-CSF in regulating tumor-associated macrophages (TAMs) and its role in modulating host immune responses. Through the specific binding to its receptor colony-stimulating factor 1 receptor (CSF-1R), M-CSF orchestrates a cascade of downstream signaling pathways to modulate macrophage activation, polarization, and proliferation. Furthermore, M-CSF extends its influence to other immune cell populations, including dendritic cells. Notably, the heightened expression of M-CSF within the tumor microenvironment is often associated with dismal patient prognoses. Therefore, a comprehensive investigation into the roles of M-CSF in tumor growth advances our comprehension of tumor development mechanisms and unveils promising novel strategies and approaches for cancer treatment.

## 1 Introduction

Cancer has emerged as a highly fatal disease on a global scale, posing a significant threat to human health and life (1–3). As medical research advances, it has become increasingly evident that the immune system plays a pivotal regulatory role in the initiation and progression of cancer. Among the various immune cells in tumor tissues, macrophages constitute the most abundant population and exert multifaceted functions in tumor onset, development, metastasis, and therapeutic responses.

Macrophages primarily serve as integral components of the human immune system, with distinct subtypes performing diverse immunological functions. Notably, M1 macrophages are responsible for identifying and eradicating cancerous cells. Their direct anti-tumor actions involve phagocytosis and destruction of malignant cells. Additionally, M1 macrophages modulate immune responses by secreting pro-inflammatory cytokines and chemical factors and influencing the activities of other immune cells, thereby stimulating the host’s immune reactions against tumors. Macrophage colony-stimulating factors (M-CSFs) are crucial in this regulatory process. As a pivotal cytokine, M-CSF exerts a multifaceted influence on macrophage behavior, enhancing phagocytic and chemotactic activities, and augmenting cytotoxicity against tumor cells.

The tumor microenvironment represents a complex ecological system, comprising tumor cells, immune cells, extracellular matrix components, and a plethora of cytokines (4). Beyond their regulatory interactions with tumor-associated macrophages (TAMs), M-CSF engages in intricate cross-talk with various other cell types within the tumor microenvironment, impacting diverse physiological processes such as immunoregulation and metabolism, thereby forming a complex regulatory network (5, 6). This intricate regulation can profoundly influence tumor growth, invasion, metastasis, and immune evasion, underscoring the far-reaching implications of M-CSF in the context of cancer.

In clinical practice, an increasing body of research has demonstrated a strong correlation between the elevated expression of M-CSF and poor patient prognosis across various types of tumors (7, 8). This association underscores the potential of M-CSF as a promising target for novel therapeutic strategies in cancer management. For instance, interventions aimed at inhibiting M-CSF or their receptor, colony-stimulating factor 1 receptor (CSF-1R), hold the potential to modulate the tumor microenvironment, thereby impeding tumor progression and offering improved survival prospects for patients. In recent years, extensive research has underscored the pivotal role of M-CSF in tumor development. However, the precise regulation mechanisms and their potential as therapeutic and predictive indicators remain focal points of current research.

This study explored the role of M-CSF in tumor development. Initially, an analysis was conducted of the structural attributes, expression patterns, and regulatory mechanisms of M-CSF and their receptor CSF-1R, specifically emphasizing their interactions with host immune responses. Subsequently, the impact of M-CSF on existing clinical therapeutic approaches was investigated, while considering the modulation strategies involving other cytokines. Lastly, a perspective was provided on the future of M-CSF in cancer research, focusing on elucidating their interactions with TAMs and tumor cells, the development of novel diagnostic and therapeutic strategies, and the potential for personalized treatments.

## 2 Gene structure and biochemical characteristics of M-CSF

M-CSF, also known as CSF-1, is a cytokine that selectively stimulates the proliferation of hematopoietic progenitor cells, guiding their specific differentiation into the mononuclear phagocyte lineage (9). As a chemotactic factor, M-CSF plays a vital role in the survival, proliferation, and differentiation of mononuclear phagocytes within hematopoietic stem cells.

**Figure 1:**
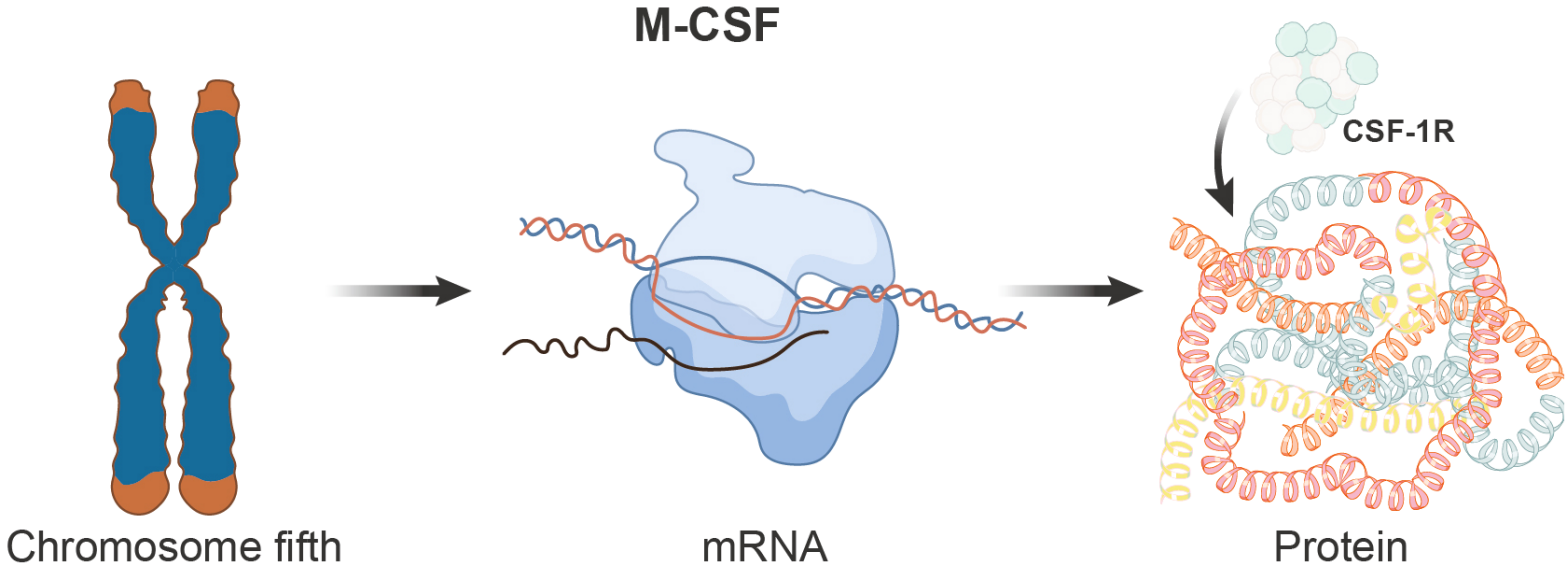
Schematic depicting the genomic, transcriptional, and translational aspects of the M-CSF gene. The M-CSF gene is located on chromosome 5 in humans and undergoes transcription to produce multiple mRNA isoforms, which subsequently translate into distinct protein isoforms. The M-CSF protein encompasses various functional domains, with the domain responsible for binding to its receptor, CSF-1R, being of paramount importance.

The gene encoding M-CSF is localized on the human chromosome 5. This gene undergoes transcription, resulting in multiple mRNA isoforms that can translate into various protein isoforms. The M-CSF protein is typically a glycoprotein modified with numerous N-glycan and glycosyl residues. These modifications are critically important for its stability and activity. Specifically, M-CSF exhibits three biologically active isoforms: secreted glycoprotein (80-100kDa), secreted protein-polysaccharide (130-160kDa), and cell surface glycoprotein spanning the cell membrane (68-86kDa). Structurally, the M-CSF protein comprises multiple functional domains, with the most crucial being the domain responsible for binding to its receptor CSF-1R (**Figure 1**).

### 2.1 Biosynthesis and cellular origins of M-CSF

The biosynthesis of M-CSF commences with the transcription of its gene. Following translation, the initial M-CSF precursor protein enters the rough endoplasmic reticulum, where it undergoes initial folding and glycosylation modifications. Subsequently, M-CSF is transported to the Golgi apparatus, where final modifications and packaging occur. In the Golgi apparatus, M-CSF acquires its ultimate glycosylation form and is packaged into vesicles, which subsequently fuse with the cell membrane, leading to the secretion of M-CSF into the extracellular space. It is noteworthy that M-CSF can be produced by various cells, including monocytes, fibroblasts, osteoblasts, activated macrophages, neurons, epithelial cells, bone marrow stromal cells, activated endothelial cells, and tumor cells. This wide range of cellular origins enables M-CSF to exert its effects under different physiological and pathological conditions.

### 2.2 Mechanisms of M-CSF in regulating host immune responses

M-CSF, a crucial cytokine in the immune system, assumes a significant role in the regulation of host immune responses. It serves as the principal regulatory factor for the survival, proliferation, and differentiation of macrophages. M-CSF promotes the survival of monocytes, their differentiation into macrophages, and subsequent macrophage proliferation. Furthermore, it initiates and enhances macrophage-mediated cytotoxicity against tumor cells and microbes, regulates the release of cytokines and other inflammatory modulators by macrophages, and stimulates phagocytosis.

Macrophages, positioned at the forefront of the immune system, assume a central role in clearing both exogenous and endogenous pathogens. M-CSF modulates the survival, proliferation, differentiation, and functions of macrophages (10–12). Particularly during the early stages of the host’s immune response, the expression and secretion of M-CSF increase, facilitating the differentiation of hematopoietic progenitor cells in the bone marrow into macrophage lineages (13, 14). Additionally, M-CSF activates a series of signaling pathways by binding to its receptor, CSF-1R, to regulate various functions of macrophages (13, 15, 16). Notably, the influence of M-CSF on macrophage polarization is of particular significance, as it drives macrophages toward an M2 polarization state, characterized by anti-inflammatory and tissue repair functions (11, 17–19).

M-CSF exerts regulatory effects not only on innate immunity but also on adaptive immunity. In certain diseases such as cancer and autoimmune disorders, M-CSF modulates T cell activation and function through its regulation of macrophage functionality (14, 18, 20, 21). This regulation is partially mediated by M2-type macrophages induced by M-CSF, which can secrete anti-inflammatory factors like interleukin (IL)-10, thereby suppressing effector T cell activation and modulating immune responses (13, 22, 23). Research indicates that M-CSF also impacts the functionality of other immune cells, such as dendritic cells (18, 24). It has been implicated in dendritic cell differentiation and development, and it can also affect T-cell activation (18). Furthermore, M-CSF regulates the survival and proliferation of NK, T, and B cells and neutrophils, further expanding its role in immune responses (25, 26).

M-CSF not only assumes a crucial role in cancer but also exerts significant regulatory effects in non-neoplastic diseases. In autoimmune conditions like rheumatoid arthritis, M-CSF may be overexpressed, correlating with disease progression and exacerbation of pathology (27–29). Furthermore, due to its influence on adaptive immunity, M-CSF also plays a regulatory role in some immune-related disorders.

## 3 Regulatory role of M-CSF on TAMs

M-CSF plays a pivotal role in regulating macrophages within the immune system, exerting a critical influence on the roles of macrophages in diseases such as cancer. Macrophages can differentiate into two primary phenotypes: M1 and M2. M1 macrophages are associated with pro-inflammatory and anti-tumor functions, while M2 macrophages are typically linked to anti-inflammatory responses, tissue repair, and tumor promotion. Within the tumor microenvironment, macrophages can differentiate into TAMs, assuming multifaceted roles in tumor development, metastasis, and immune evasion. The influence of M-CSF on macrophage differentiation is particularly noteworthy. It tends to promote the differentiation of macrophages towards the M2 phenotype, which is associated with tumor-promoting properties (11). This M2 polarization, driven by M-CSF, supports tumor growth by suppressing anti-tumor immune responses and promoting tissue remodeling and angiogenesis, which are beneficial for tumor progression (30). The regulation of M-CSF and its impact on macrophage differentiation highlight the complex interplay between the immune system and cancer, underscoring the potential of M-CSF as a therapeutic target in cancer treatment (31).

### 3.1 Dual Role of TAMs in tumor microenvironment and their immune regulatory mechanisms

**Figure 2:**
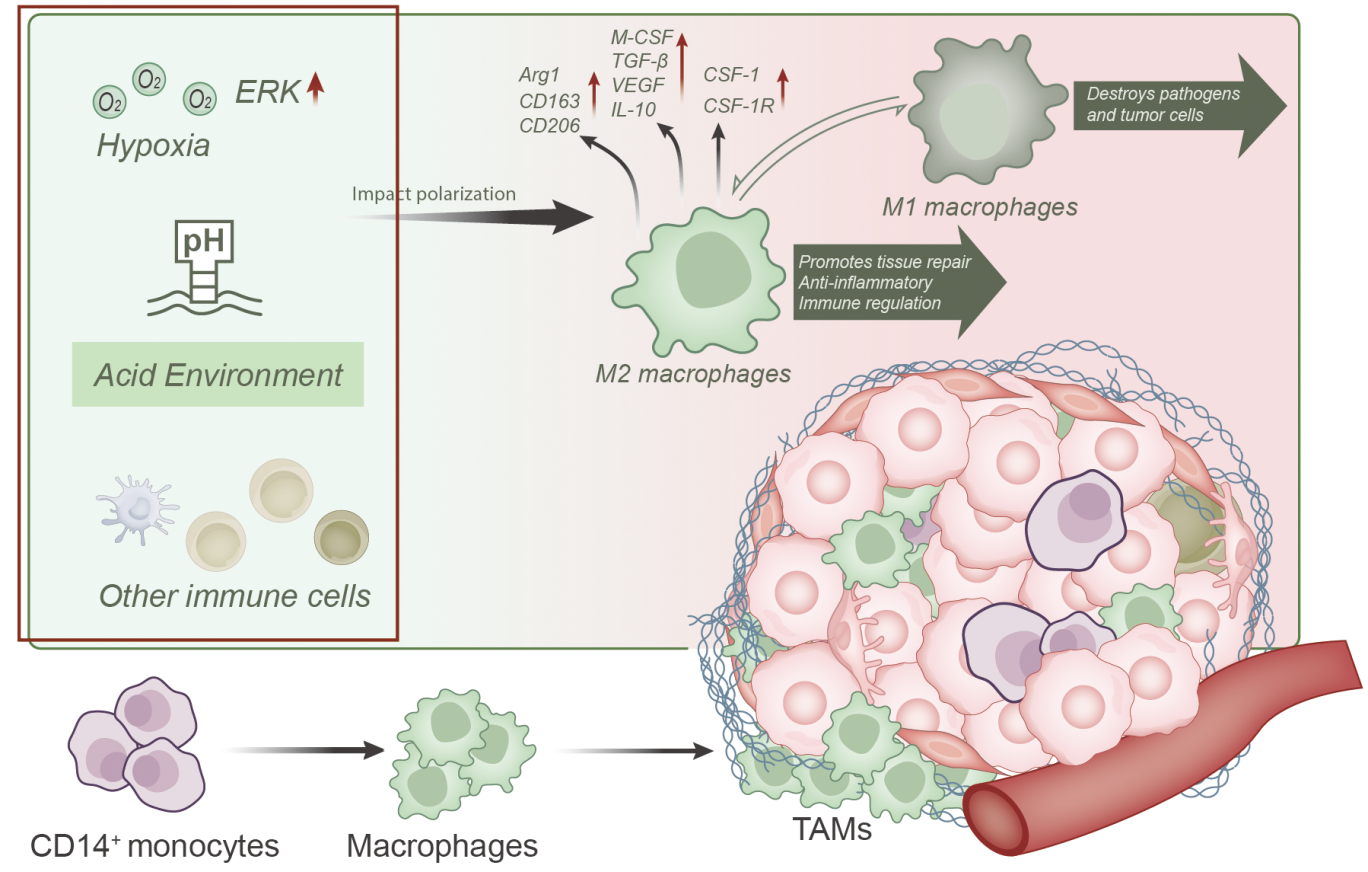
Overview of TAMs differentiation, function, and factors influencing their polarization within the tumor microenvironment. TAMs predominantly originate from CD14+ monocytes in the bone marrow and differentiate into macrophages within the tumor microenvironment. Various influencing factors, such as hypoxia, acidic conditions, and interactions with other immune cells, play a role in polarizing TAMs toward an M2-like phenotype. M2-like TAMs are characterized by elevated expression of markers like Arg1, CD163, and CD206, along with the production of immunosuppressive cytokines like M-CSF, TGF-β, VEGF, and IL-10. Overexpression of CSF-1 and CSF-1R on M2 macrophages is a key contributor to unfavorable tumor prognosis.

TAMs represent a prominent immune cell population within the tumor microenvironment, exerting pivotal roles in tumor development and metastasis (**Figure 2**) (17, 18, 32–36).

TAMs primarily originate from CD14^+^ monocytes from the bone marrow and differentiate into macrophages upon entering the tumor microenvironment (11, 13, 14, 16, 23), where they subsequently undergo local proliferation (13). TAMs dominate the tumor microenvironment (17, 18, 21, 34), particularly in the peritumoral regions, outnumbering intratumoral macrophages (23) and accounting for approximately 40% of total tumor cell population (35).

TAMs are typically categorized into two subtypes, known as the classically activated phenotype (M1 or M1-like phenotype) and alternatively activated phenotype (M2 or M2-like phenotype) (12, 14, 22, 23, 35, 37–39). Importantly, these M1 and M2 macrophages represent dynamically interconvertible subpopulations, and their phenotypes can reversibly switch (11, 12, 40). Consequently, TAMs exhibit a dualistic nature, capable of both inhibiting and promoting tumor development (11). In their tumor-suppressive role, TAMs engulf tumor cells and apoptotic cells while producing cytotoxic oxygen and nitrogen species, cytokines, and enzymes to restrain tumor growth (40, 41). Conversely, more recent research findings have highlighted TAMs’ pro-tumorigenic effects (12, 34), as they support tumor growth and metastasis through various mechanisms (36, 42), including promoting angiogenesis, suppressing anti-tumor immune responses (13, 22, 23), facilitating tumor cell invasion and metastasis (11, 21), and sustaining the survival of tumor stem cells (21).

In the tumor microenvironment, TAMs typically exhibit characteristics of M2 macrophages, which are associated with functions such as tissue repair promotion, anti-inflammatory responses, and immune regulation (12, 13, 17, 37, 42). These cells are characterized by the high expression of arginase-1 (Arg1), scavenger receptor CD163, and mannose receptor CD206 (42, 43). Importantly, these M2-like TAMs rely on cellular bioenergetic pathways, including the tricarboxylic acid (TCA) cycle, fatty acid oxidation (FAO), glycolysis, and mitochondrial oxidative phosphorylation (OxPhos) as their primary sources of cellular energy (22, 44). Furthermore, these M2-like TAMs produce various immunosuppressive cytokines such as M-CSF, transforming growth factor-β (TGF-β), vascular endothelial growth factor (VEGF), and IL-10, among others, which are categorized as Th2 cell-associated cytokines (38). These cytokines can promote tumor growth, immune evasion, and angiogenesis (13, 20, 22, 37). TAMs are frequently found in cancerous tumors, and their abundance correlates with poor prognosis (42). Overexpression of CSF-1 and CSF-1R on M2 macrophages represents a significant factor contributing to adverse tumor prognosis (19).

However, it is worth noting that the polarization state of TAMs is highly adaptable and influenced by various factors within the tumor microenvironment. For instance, hypoxic conditions can promote M2-like phenotype polarization by activating the ERK signaling pathway, thereby facilitating tumorigenesis (45). Additionally, factors such as the acidic conditions in the tumor microenvironment, factors secreted by tumor cells, and interactions with other immune cells are crucial determinants influencing TAM polarization (11, 13, 14, 33, 38, 46–49). These factors collectively form a complex regulatory network that dictates the multifaceted roles of TAMs in tumor development and immune evasion.

### 3.2 Signaling pathways mediated by M-CSF and their key role in TAMs

**Figure 3:**
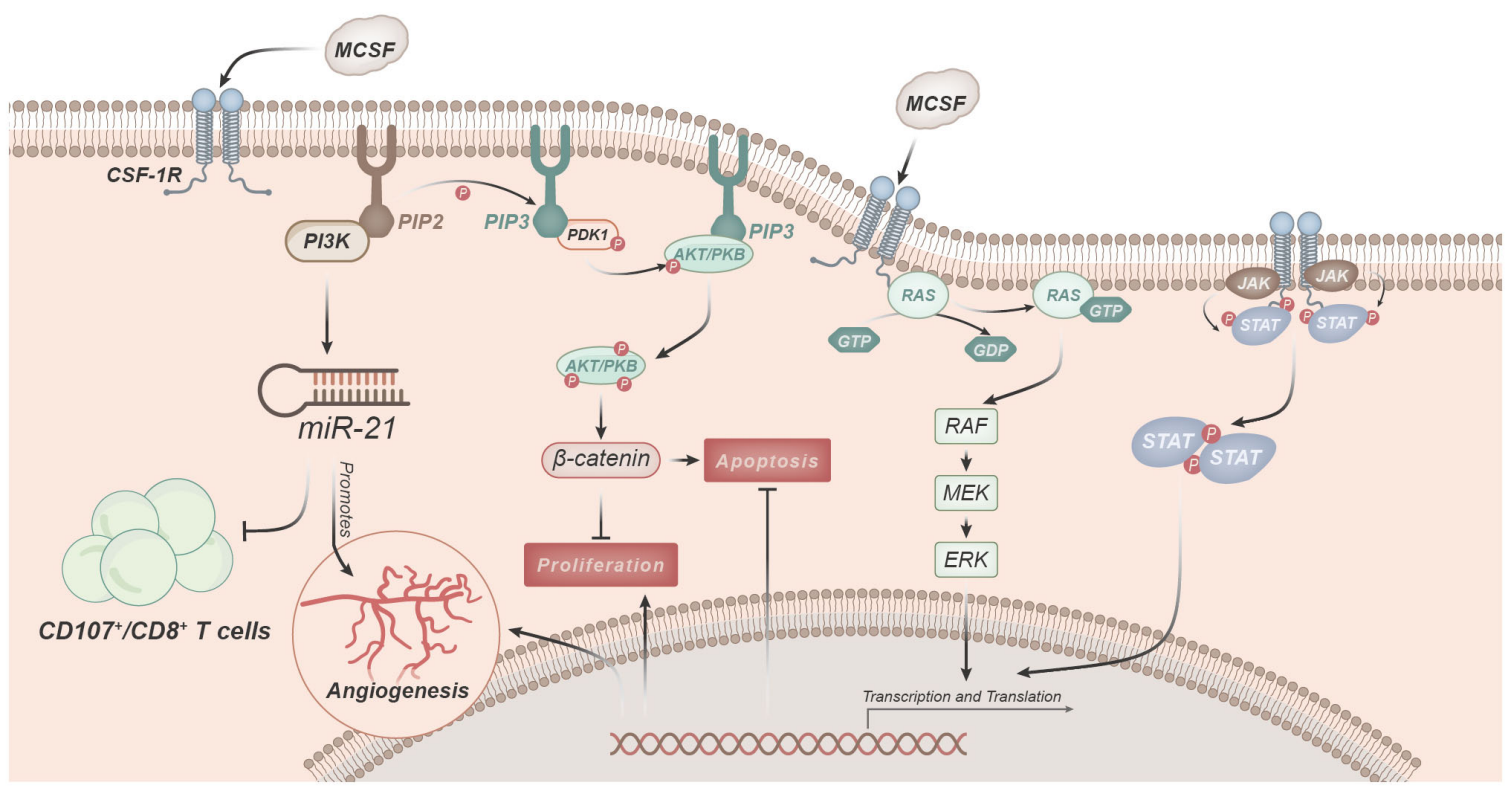
Signaling pathways involved in macrophage regulation and function mediated by M-CSF. M-CSF predominantly modulates macrophage functions by binding to its receptor, CSF-1R. Activation of CSF-1R triggers the PI3K pathway, leading to the conversion of PIP2 to PIP3. Subsequently, PIP3 facilitates the phosphorylation and activation of Akt kinase. Activated Akt, in turn, regulates various cellular functions, including macrophage survival, proliferation, and anti-apoptosis. CSF-1R signaling, through PI3K activation, induces miR-21 expression, promoting angiogenesis. Downstream signaling cascades initiated by CSF-1R, such as the Ras-Raf-MEK-ERK pathway, activate nuclear transcription factors, thereby initiating gene expression.

M-CSF regulates macrophage functions primarily by binding to its receptor CSF-1R and initiating downstream signaling pathways (**Figure 3**) (33).

One of the key pathways involved is the PI3K-Akt pathway (11, 40, 47). Upon stimulation by M-CSF, activation of CSF-1R leads to the activation of PI3K, which catalyzes the conversion of phosphatidylinositol 4,5-bisphosphate (PIP2) to phosphatidylinositol 3,4,5-trisphosphate (PIP3). PIP3, in turn, promotes the phosphorylation and activation of Akt kinase (33). Activated Akt leads to the phosphorylation of GSK 3β, resulting in the release of β-catenin, which can mediate the regulation of various cellular functions (10, 24, 32, 33). CSF-1R signaling induces the expression of miR-21 through the activation of PI3K, thereby promoting angiogenesis. Simultaneously, this signal inhibits immune responses, such as reducing the number of CD107/CD8^+^ T cells in TAMs (11, 13, 14, 18, 36). Furthermore, transcriptional analysis also indicates the critical role of the PI3K-Akt signaling pathway in M-CSF-mediated regulation of macrophage functions (11).

Subsequently, the MAPK pathway comes into play. Downstream signaling pathways activated by CSF-1R, such as the Ras/Raf/MEK/ERK pathway, can activate nuclear transcription factors and initiate gene expression (11, 40). Macrophages activated by ERK are involved in cell growth, proliferation, differentiation, and inflammation regulation (14, 24, 32, 33, 50, 51). The MAPK/ERK pathway ultimately leads to an increase in the expression of biologically active VEGF, a key factor in the process of neovascularization. This regulation of VEGF primarily occurs at the gene transcription level, promoting the production of VEGF by monocytes and macrophages (52).

Moreover, the JAK-STAT pathway is also a crucial signaling pathway in M-CSF-mediated regulation of macrophage functions (32). Upon binding of M-CSF to CSF-1R, it activates JAK protein kinases, leading to their phosphorylation and activation. Subsequently, activated JAK further phosphorylates and activates STAT proteins. Phosphorylated STATs form homodimers or heterodimers that translocate to the cell nucleus, thereby controlling gene transcription and expression. This axis is a core intracellular signaling pathway that regulates various cytokine and growth factor signaling cascades, mediating downstream events such as inflammation infiltration, immune responses, repair, proliferation, differentiation, movement, and cell apoptosis (53).

In addition to the aforementioned signaling pathways, M-CSF can also regulate macrophage functions through pathways like NF-κB (54). The activation and interaction of these signaling pathways collectively regulate macrophage proliferation, survival, and polarization, enabling them to play a role in inflammation and immune responses.

In summary, the activation and interaction of these signaling pathways reflect the crucial role of macrophages in the immune system, not only in inflammatory responses but also in understanding their role in immune responses and diseases.

### 3.3 Regulatory role of M-CSF on TAM functions

M-CSF, as a pivotal regulatory molecule, confers a paramount role in the tumor microenvironment’s macrophages. Its influence extends beyond mere cellular survival and death, encompassing how these cells respond to stimuli from tumor cells. M-CSF exerts critical control over TAMs. This regulation is orchestrated through various molecular mechanisms, ensuring that macrophages exhibit functionality and adaptability within the tumor microenvironment. Furthermore, M-CSF provides the essential survival signals required by TAMs. This signaling cascade guarantees the proper protection of macrophages within the tumor microenvironment, averting premature cell death. In summary, M-CSF imparts crucial effects in regulating the fundamental functions of TAMs.

M-CSF exerts its effects not only through its intrinsic activity but also by activating various intracellular signaling pathways. Among these pathways, the PI3K-Akt signaling pathway serves as the principal avenue, responsible for promoting the survival, proliferation, anti-apoptosis, and migration of TAMs (55, 56). Additionally, M-CSF further refines its control over cells by activating the MAPK and JAK-STAT pathways (57, 58). These signaling pathways interact intricately within cells, forming a complex network that ensures cellular stability within the tumor environment. Particularly in the early stages of tumor progression, M-CSF, through these pathways, assures the survival and functionality of macrophages. In conclusion, M-CSF regulates the functionality of TAMs through multiple signaling pathways.

The tumor microenvironment represents a complex cellular network in which M-CSF serves as a bridging element. This bridge connects macrophages with tumor cells, leading macrophages to preferentially differentiate into M2-type macrophages within the tumor environment. These macrophages exhibit characteristics of immunosuppression and anti-inflammatory responses, further promoting tumor growth and development. Moreover, the overexpression of M-CSF reinforces this tendency, resulting in TAMs displaying more pronounced immunosuppressive traits. This feature is considered a crucial mechanism for tumor immune escape surveillance. In summary, the influence of M-CSF in the tumor microenvironment is both intricate and far-reaching.

Immunomodulation is another crucial role of M-CSF in the tumor microenvironment. M-CSF balances the degree of inflammatory reactions by regulating the production and release of inflammatory factors within macrophages (59, 60). For instance, it can inhibit the production of TNF-α and IL-6, thereby alleviating inflammatory responses. Simultaneously, M-CSF enhances macrophages’ capacity for inflammatory responses, enabling them to produce more lysozymes and oxygen radicals. This bidirectional action underscores the complexity of the role of M-CSF in immunomodulation. Therefore, M-CSF exerts a bidirectional and intricate role in regulating TAMs’ inflammatory responses.

Apart from the aforementioned primary functions, M-CSF has additional roles that are equally significant within the tumor microenvironment. For instance, M-CSF can induce macrophages to express certain immunosuppressive molecules, such as PD-L1 and PD-L2, contributing to the attenuation of macrophages’ anti-tumor activity (61). Moreover, M-CSF can regulate the formation of immune synapses in TAMs, which constitutes a critical interaction between immune cells and tumor cells. This interaction influences intercellular signal transduction and effector responses, further impacting tumor development. In summary, M-CSF assumes an indispensable role in regulating various functions of TAMs.

## 4 Impact of M-CSF on tumor development

Macrophage infiltration confers a direct or indirect regulatory role in the formation and progression of many tumors. In particular, the regulation of TAMs by M-CSF provides a favorable microenvironment for tumors, thus influencing tumor growth, metastasis, and immune evasion. Furthermore, the interactions between M-CSF and other cells and molecules in the tumor microenvironment also drive tumor progression. Understanding these effects of M-CSF provides us with a deeper insight and aids in the development of novel therapeutic strategies aimed at intervening in the tumor microenvironment, ultimately aiming to inhibit tumors or enhance the effectiveness of immunotherapy.

### 4.1 Catalytic role of M-CSF in tumor progression

With the advancement of scientific research, it has been firmly established that M-CSF plays a pivotal role in the field of tumor biology, particularly in the processes of tumor initiation, progression, and metastasis. The expression of M-CSF is intricately linked to tumor growth and development.

On the one hand, both tumor cells and other cells within their microenvironment, such as TAMs and tumor-associated dendritic cells, release various growth factors, including M-CSF, MCP-1, MCP-2, macrophage inflammatory protein-1α, and macrophage inflammatory protein-1β. Collectively, these cytokines drive tumor proliferation and growth by promoting the survival and proliferation of tumor cells, supplying them with essential nutrients, and further facilitating their migration and metastasis. In order to create a more favorable growth environment for tumor cells, it has been observed that tumor cells can produce M-CSF, consequently guiding the accumulation of surrounding tissue cells towards the tumor. This process may involve epithelial-to-mesenchymal transition (EMT), which enhances the migratory and invasive capabilities of tumor cells. Specifically, M-CSF can activate a series of transcription factors, such as Snail, Slug, and Twist, which inhibit the expression of the cell adhesion protein E-cadherin, thereby regulating the EMT process (62). Moreover, M-CSF can modulate the expression of cell cycle-related proteins within tumor cells, further driving their proliferation (63). On the other hand, M-CSF, through its binding with its specific receptor CSF-1R, activates multiple critical downstream signaling pathways such as PI3K/AKT and MAPK/ERK, thereby promoting tumor cell migration (64).

However, the impact of M-CSF extends beyond tumor cells alone. It plays a crucial role in the regulation of immune cells within the tumor microenvironment. For instance, M-CSF can enhance the proliferation of tumor-associated dendritic cells while reducing their activation and antigen-presenting capacity, leading to functional impairments and an inability to induce the proliferation of tumor-specific CD4^+^ and CD8^+^ T cells. Consequently, this affects the recognition and clearance of tumors by other immune cells. This also explains the close association between M-CSF expression levels and the degree of immune infiltration in tumors, patient prognosis, and treatment responsiveness.

In summary, M-CSF exerts multifaceted effects on the formation and progression of tumors. It not only directly influences tumor cells but also modulates immune cells within the tumor microenvironment and impacts the secretion of relevant molecules by immune cells, ultimately affecting tumor growth.

### 4.2 Regulatory mechanism of M-CSF on TAM-mediated tumor cell proliferation

Tumor development is a complex biological process involving interactions among various cell types. Particularly within the tumor microenvironment, M-CSF exerts a significant influence on tumor cell proliferation through the regulation of TAMs.

M-CSF not only directly stimulates the proliferation of liver cancer cells (65). More importantly, it plays a pivotal role in regulating TAMs. Multiple studies have confirmed that M-CSF can induce TAMs to differentiate into M2-type macrophages (66). In comparison to traditional M1-type macrophages, M2-type macrophages exhibit unique roles in immune modulation, primarily characterized by their anti-inflammatory and immunosuppressive properties. These cells release immunosuppressive factors such as TGF-β and IL-10, which can inhibit the activity of cytotoxic T cells, thus creating a more favorable growth environment for tumor cells.

Furthermore, M-CSF engages in close interactions with tumor cells and other cells within their microenvironment. For instance, tumor cells can produce M-CSF, attracting and guiding surrounding macrophages to accumulate at the tumor site, collectively creating a microenvironment conducive to tumor growth. These recruited TAMs, in turn, release more M-CSF, binding to CSF-1R on the surface of tumor cells, forming a positive feedback loop that facilitates continuous growth and survival of tumor cells.

### 4.3 Role of M-CSF in TAM-mediated tumor cell migration

The migration and infiltration of tumor cells are critical factors in tumor development and metastasis. Therefore, it is imperative to explore the role of M-CSF and its regulation of TAMs in this process.

In co-culture models of macrophages and tumor cells, macrophages predominantly exhibit M2-like characteristics. These macrophages not only promote the EMT process of colorectal cancer cells, enhancing their migratory and invasive capabilities (63), but also indirectly regulate the migration of tumor cells through various mechanisms. M-CSF can induce TAMs to differentiate towards the M2 phenotype (14, 17), thereby promoting the migration of tumor cells by secreting various cytokines such as epidermal growth factor (EGF), TNFs, and ILs (38). Tumor cells also self-secrete M-CSF, which not only attracts nearby macrophages to gather at the tumor site but also stimulates macrophages to release more M-CSF, binding to CSF-1R on the surface of tumor cells, forming a potent positive feedback mechanism (32, 67). Through the paracrine loop involving CSF-1R and epidermal growth factor, this mechanism enhances the interaction between tumor cells and macrophages, further promoting the migration and infiltration of tumor cells.

Hence, M-CSF holds a pivotal role in the regulation of tumor cell migration and infiltration. Its influence extends beyond direct effects on tumor cells, as it also modulates the behaviors of macrophages within the tumor microenvironment. This novel mechanistic understanding offers valuable insights and potential therapeutic targets for the treatment of cancer.

### 4.4 Interactions of M-CSF-mediated TAMs with tumor cell signaling pathways

The interaction between M-CSF and its receptor CSF-1R holds a central position in regulating the tumor microenvironment, particularly in the context of signaling crosstalk between TAMs and tumor cells.

Firstly, M-CSF influences the communication between TAMs and tumor cells by promoting TAM differentiation and proliferation (13, 14, 16). TAMs are a major immune cell type within the tumor microenvironment and play crucial roles in tumor growth, invasion, and metastasis (36). M-CSF, through binding to its receptor CSF-1R, not only activates signaling pathways such as PI3K/Akt and MAPK, driving TAM survival and proliferation, but also induces TAM polarization towards the immunosuppressive M2-like macrophages, reinforcing their supportive role in promoting tumor cell growth (18, 21, 67).

Secondly, M-CSF modulates TAM cytokine secretion, including IL-2, VEGF, EGF, TGF-β, and CCL22 (13, 21, 68), thereby impacting the behavior of tumor cells. These cytokines play diverse roles in tumor development, including stimulating angiogenesis, suppressing T cell-mediated immune responses, and promoting tumor cell survival and proliferation (13, 23, 36). Particularly under M-CSF regulation, TAMs promote angiogenesis and tumor cell proliferation by secreting VEGF and EGF, creating a favorable microenvironment for tumor progression (22).

Furthermore, M-CSF also regulates the expression of surface molecules on TAMs, such as PD-L1 and CD206, enhancing the signaling crosstalk between TAMs and tumor cells. The expression of PD-L1, an immune checkpoint molecule, on TAMs inhibits the activity of CD8^+^ T cells, creating favorable conditions for tumor growth and metastasis. Additionally, CD80 and CD86 as co-stimulatory molecules are expressed on TAMs, influencing immune responses in the tumor microenvironment by regulating T cell activation and responses (20, 21, 36). CD206, as a marker of M2-like macrophages, further supports the polarization of TAMs towards the M2-like phenotype following M-CSF action (36).

Therefore, M-CSF exerts its influence on the tumor microenvironment through multiple mechanisms to modulate TAM behavior, subsequently affecting tumor cell growth, migration, and infiltration. This finding provides a novel theoretical foundation and strategic direction for immunotherapy approaches against cancer.

## 5 Clinical applications of M-CSF: therapeutic strategies and outcomes

Given the pivotal role of M-CSF in tumor development, it has become a focal point of research in cancer treatment strategies. The following sections delve into the clinical applications of M-CSF and how its activity can be modulated by other cytokines to enhance treatment outcomes for patients.

### 5.1 Characteristics of M-CSF in tumor tissues

The role of M-CSF in tumor tissues is often significant, playing a critical role in tumor progression. High expression of M-CSF has been observed in various cancer types, including breast cancer, lung cancer, gastric cancer, and colorectal cancer (15, 32). Furthermore, studies have found that M-CSF expression correlates with disease severity and stages in ovarian cancer, glioblastoma, and triple-negative breast cancer (TNBC) (38, 46, 69). In recent years, a plethora of research has shown that M-CSF expression increases with the growth of tumor tissues. For instance, a previous study has noted that M-CSF expression is higher in lung cancer at advanced stages than at early stages (38, 70), and elevated M-CSF levels in the serum of patients with various types of cancer, including pancreatic and cervical cancers, were associated with advanced-stage tumors (71, 72). High levels of M-CSF expression were also significantly correlated with the distribution of CD163/CD68 TAMs in grade IV glioblastomas (38, 69). Furthermore, TNBC secretes more M-CSF compared to non-TNBC (46).

High M-CSF expression is not limited to tumor cells alone. In the tumor microenvironment, M-CSF is also produced by other cells, such as TAMs and stromal cells (38). These cytokines significantly impact the growth, differentiation, and functional expression of macrophages by binding to their specific receptor, c-fms (14). Additionally, M-CSF can regulate TAM polarization, promoting their transformation into an M2-like phenotype, further supporting tumor growth and metastasis (18).

It is worth noting that M-CSF is not only associated with tumor growth and metastasis but also with challenging aspects of cancer treatment, such as immune evasion, drug resistance, and radiotherapy resistance. Therefore, research on M-CSF not only aids in gaining a deeper understanding of the biological characteristics of tumors but may also provide new strategies and approaches for cancer treatment.

### 5.2 Correlation analysis of the role of M-CSF with clinical prognosis in cancer patients

In the context of cancer treatment and management, the identification and assessment of prognostic factors are of paramount importance. These factors not only offer predictions about potential clinical outcomes for patients but also provide essential information for tailoring individualized treatment plans. In this context, future research should pay attention to the functional relevance of M-CSF in the tumor microenvironment, particularly its regulation of TAMs.

High expression of M-CSF has been linked to adverse clinical outcomes in various cancer types (32, 69). Notably, in non-small cell lung cancer, studies have revealed a positive correlation between M-CSF expression and the degree of TAM infiltration, which, in turn, significantly correlates with 5-year overall survival and disease-free survival rates. Specifically, patients with high M-CSF expression have a markedly lower 5-year overall survival rate of only 19.6%, compared to 51.3% in patients with lower expression levels (11, 38, 73).

The composition of immune cells within the tumor microenvironment, especially the presence and functional status of TAMs, provides crucial clues for assessing tumor progression. Immunological and stromal scores aid in evaluating the extent of immune cell infiltration and tumor purity in the tumor microenvironment (69). TAMs, particularly M2-like macrophages, are directly associated with the regulation of M-CSF and the release of other factors. M2-like macrophages are believed to exert inhibitory effects on the anti-tumor immune response. Prior research has indicated that TAMs are the predominant inflammatory cells in various cancers and are closely linked to disease progression and prognosis (73). Cytokines derived from M2 macrophages, such as IL-10 and TGF-β, are considered major mechanisms for suppressing anti-tumor immune activity (14).

In summary, the interplay between M-CSF and TAMs, especially M2-like macrophages, significantly impacts the immune microenvironment of tumors and patient prognosis. Clinical research has demonstrated the association of high M-CSF expression with adverse outcomes. Therefore, a thorough exploration of the role of M-CSF is crucial for improving treatment strategies and prognostic assessments.

### 5.3 Role of M-CSF in clinical therapies and strategies for modulation

M-CSF has been recognized as a crucial therapeutic target in cancer treatment (5, 38, 46). Multiple studies have demonstrated that inhibiting the interaction of CSF-1/CSF-1R, leading to the elimination of TAMs, is an effective strategy in cancer therapy (5, 11, 20, 21, 24, 32). Inhibitors targeting M-CSF or its receptor c-fms have shown significant effects in suppressing tumor growth and improving prognosis (20, 34). For instance, daily treatment with an anti-CSF-1R therapeutic antibody (50 mg/kg) has been proven to reduce the number of macrophages in a peritumoral osteosarcoma mouse model. Axatilimab, a novel humanized monoclonal antibody, can block the binding of M-CSF to its receptor, thereby inhibiting CSF-1R signaling and macrophage development (74). Moreover, active components of traditional Chinese medicine, such as cannabidiol, have been found to inhibit M-CSF secretion by melanoma cells, thereby affecting the immunosuppressive tumor microenvironment (49). Thus, the targeting of M-CSF in cancer therapy and the application of its inhibitors in combination therapy provide a promising and novel strategy for clinical cancer treatment.

M-CSF has been extensively reported to control immune responses, particularly processes related to tumor immune responses. It can stimulate the production and activation of M2 macrophages, resulting in a decrease in the number of antigen-presenting cells within the tumor microenvironment. Furthermore, M-CSF can inhibit macrophage phagocytosis and cytotoxicity, further reducing the body’s attack on tumor cells. Therefore, the regulatory role of M-CSF may impact the effectiveness of tumor treatment. When macrophage functions are suppressed, the body’s ability to attack tumor cells is diminished. Hence, understanding and manipulating the role of M-CSF in tumor immune responses are crucial for the development of more effective cancer treatment strategies.

M-CSF is closely associated with the process of angiogenesis in tumors. Research indicates that M-CSF can enhance the angiogenic capacity of tumor cells, thereby providing more blood supply and nutrients to the tumor (13, 23). M-CSF achieves this effect by inducing the survival of vascular endothelial cells and promoting the expression of factors related to angiogenesis, such as VEGF (75). In a mouse osteosarcoma model, inhibiting M-CSF activity selectively suppressed tumor angiogenesis and lymphangiogenesis, offering possibilities for exploring novel treatment strategies (76).

M-CSF is involved in the recruitment of macrophages in the tumor microenvironment (13, 20, 77). This process is crucial for sustaining tumor growth and metastasis. M-CSF antibodies have been demonstrated to significantly inhibit macrophage migration, thereby reducing the content of F-actin (46).

Although M-CSF and its receptor inhibitors have shown some effectiveness in cancer treatment, combining them with other treatment methods is more likely to yield significant therapeutic benefits. For example, PLX3397 is a known M-CSF receptor inhibitor that not only depletes TAMs but also reduces FOXP3^+^ regulatory T cells, promoting the migration and infiltration of CD8^+^ T cells into the tumor (78). Research has also found that the combination of CSF1R inhibitors with anti-PD-L1 therapy is more effective in preventing tumor growth than single treatments (18).

M-CSF is closely related to chemotherapy and radiotherapy. Chemotherapeutic drugs, such as cyclophosphamide (CTX), have been shown to reduce M-CSF levels in tissues, thereby inhibiting tumor cell survival (68). Furthermore, M-CSF is associated with the response to radiotherapy, as the expression of M-CSF in glioblastoma cells is related to resistance to the anti-tumor drug 5-FU (38).

M-CSF assumes a substantial role in alleviating the side effects of cancer treatment. Since cancer treatments may lead to bone marrow suppression and immune function decline, the use of M-CSF can promote the proliferation and differentiation of hematopoietic stem cells, thereby improving bone marrow function and immune function. For example, the use of hydrolyzed protein injection upregulated the expression of IL-2 and M-CSF, altering the composition of bone marrow hematopoietic cell populations, increasing the numbers of hematopoietic stem cells, B lymphocytes, macrophages, and granulocytes (68). Therefore, the use of M-CSF can alleviate the side effects during treatment and improve the quality of life for patients.

M-CSF affects the interactions between immune cells and tumor cells in the tumor microenvironment and the efficacy of chemotherapy and radiotherapy. It is worth noting that while the use of M-CSF or its receptor inhibitors alone has shown therapeutic potential in some cases, more research suggests that combining them with other treatment methods may enhance therapeutic efficacy. Additionally, the application of M-CSF contributes to alleviating side effects associated with cancer treatment and improving patients’ quality of life. Therefore, in-depth research into the role of M-CSF in tumor biology and its potential as part of comprehensive treatment strategies is of paramount importance.

### 5.4 Research on cytokine-mediated regulation of M-CSF

After extensive investigation into the role of M-CSF in cancer development, researchers have acknowledged the potential interactions between M-CSF and other cytokines, sparking exploration into the potential implications of these interactions for cancer treatment. The related research has yielded novel treatment strategies and expanded our perspective on cancer research.

The utilization of strategies involving other cytokines to modulate M-CSF presents innovative avenues for cancer treatment. For example, IFN-γ has the potential to not only disrupt the signaling of M-CSF, inhibiting tumor growth, but also enhance the efficacy of various anti-tumor therapies (79). Additionally, the anti-inflammatory properties of Interleukin-10 (IL-10) are also related to its ability to inhibit M-CSF, potentially opening up new avenues for its application in cancer treatment (36). TNF-α and IL-1β, as inflammatory cytokines, play a crucial role in regulating the expression of M-CSF. They may collaboratively participate in modulating the tumor microenvironment, thereby influencing the effectiveness of immunotherapy. However, these interactions are not always beneficial, especially in the presence of M-CSF, as TNF-α may promote the proliferation of macrophages and tumor cells (11). TGF-β and EGF play important roles in regulating M-CSF. They can alter the tumor microenvironment and potentially synergize with M-CSF, either enhancing or inhibiting the effectiveness of cancer treatment.

In summary, the interactions between M-CSF and other cytokines provide a new perspective for gaining a deeper understanding of the mechanisms of cancer development and developing novel treatment strategies.

## 6 Future perspectives in M-CSF research

As our understanding of the role of M-CSF in tumor development grows, we are recognizing its pivotal involvement in regulating interactions between immune cells and tumor cells. M-CSF not only directly affects tumor cell proliferation, migration, and invasion but also exerts influence on immune cells within the tumor microenvironment, such as by promoting the polarization of macrophages and inducing an inhibitory phenotype. Furthermore, the interactions of M-CSF with signaling molecules like the Wnt, Notch, and Hedgehog pathways may further modulate tumor progression. Consequently, a thorough examination of the intricate interplay between M-CSF and these pathways, along with a systematic investigation into their role in orchestrating tumor progression, presents substantial opportunities for the identification of novel cancer therapy strategies.

Emerging technologies, such as single-cell sequencing, artificial intelligence, microfluidic chips, tumor organoid cultures, and *in vivo* imaging techniques are revolutionizing M-CSF research. For instance, microfluidic chip technology can simulate cell-to-cell interactions in the tumor microenvironment, aiding our better understanding of how M-CSF affects cellular behavior. *In vivo* imaging techniques can monitor the expression and distribution of M-CSF in animal models, providing critical information for drug development and assessment.

The potential of whole-genome sequencing to unveil genetic and epigenetic characteristics of tumors, when combined with data on M-CSF expression patterns, paves the way for personalized treatment regimens. These tailored approaches aim to maximize therapeutic benefits for each patient, emphasizing the importance of individualized care in the fight against cancer.

Moreover, the integration of M-CSF inhibitors with other treatment modalities, such as radiation therapy, immunotherapy, and other targeted therapies, such as EGFR inhibitors, BRAF inhibitors, and mTOR inhibitors, holds promise. The concurrent inhibition of M-CSF and pathways like EGFR, for instance, might synergistically enhance treatment efficacy, offering new hope for more effective cancer treatments.

While early clinical trials have shown some therapeutic efficacy of M-CSF inhibitors, there is a pressing need for further research. It is crucial to determine the optimal dosage, treatment duration, and combination strategies with other therapies. Given the variations among patients, such as genetic polymorphisms, tumor heterogeneity, and immune status, personalized treatment plans need to be developed to ensure that each patient attains the best therapeutic outcomes. Meanwhile, challenges such as drug resistance, side effects, and the potential impact on normal physiological processes due to M-CSF inhibition must not be overlooked. Future research must strive to balance treatment efficacy with minimizing side effects, ensuring not only the effectiveness of therapy but also the quality of life for patients.

**Figure 4:**
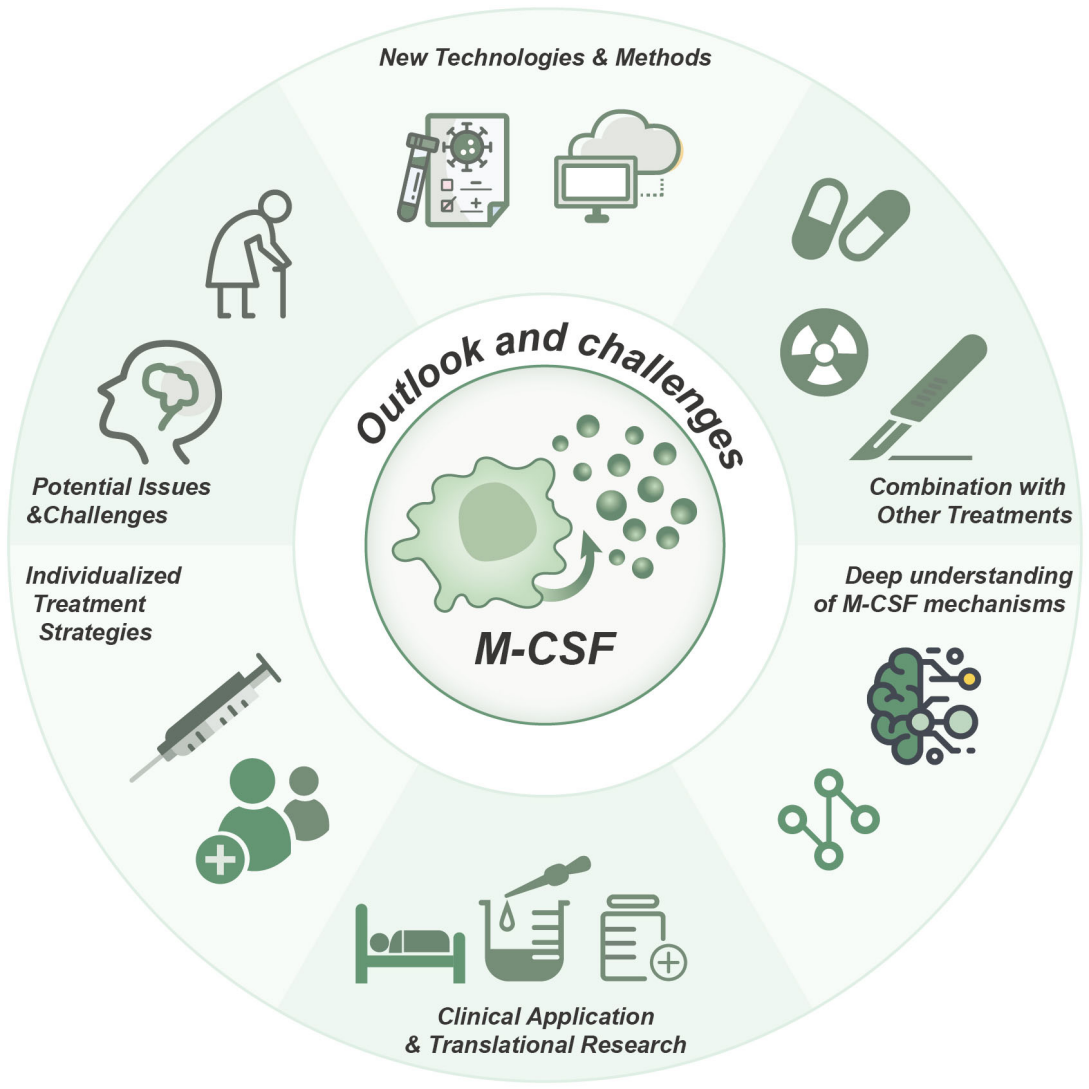
Role, strategies, and future prospects of M-CSF in cancer therapy. Targeting M-CSF as a therapeutic approach in cancer, particularly in combination therapies, holds significant promise for clinical applications. The development of individualized treatment strategies, a more profound comprehension of M-CSF mechanisms, and the utilization of innovative technologies are essential for achieving optimal therapeutic outcomes.

In conclusion, the advances in our understanding of the mechanism of M-CSF and the application of new technologies offer promising prospects for M-CSF-related strategies in oncology. The integration of M-CSF-related target therapies with other treatment modalities, the implementation of clinical applications and translational research, the formulation of personalized treatment strategies, and the resolution of potential issues and challenges emerge as crucial avenues for further investigation (**Figure 4**). Through continuous dedication and efforts, M-CSF-related target therapies holds the promise of ushering in a more optimistic future for cancer patients.

## Author contributions

LY: Conceptualization, Methodology, Writing – original draft, Writing – review & editing. YG: Conceptualization, Formal analysis, Methodology, Writing – original draft, Writing – review & editing. ZC: Data curation, Supervision, Writing – review & editing. KT: Data curation, Formal analysis, Methodology, Writing – review & editing. PL: Data curation, Supervision, Writing – review & editing. HL: Data curation, Investigation, Software, Writing – review & editing. XX: Data curation, Formal analysis, Supervision, Writing – review & editing. QP: Data curation, Investigation, Writing – original draft, Writing – review & editing. XL: Conceptualization, Formal analysis, Funding acquisition, Methodology, Project administration, Writing – original draft, Writing – review & editing.
